# Nudge interventions to reduce fish sauce consumption in Thailand

**DOI:** 10.1371/journal.pone.0238642

**Published:** 2020-09-08

**Authors:** Manasigan Kanchanachitra, Chalermpol Chamchan, Churnrurtai Kanchanachitra, Kanyapat Suttikasem, Laura Gunn, Ivo Vlaev

**Affiliations:** 1 Institute for Population and Social Research, Mahidol University, Nakhon Pathom, Thailand; 2 Department of Public Health Sciences, College of Health and Human Services, University of North Carolina at Charlotte, Charlotte, North Carolina, United States of America; 3 School of Public Health, Imperial College London, London, United Kingdom; 4 Warwick Business School, University of Warwick, Coventry, United Kingdom; Chiang Mai University Faculty of Medicine, THAILAND

## Abstract

High sodium consumption is one of the four major risk factors contributing to non-communicable diseases around the world. Thailand has one of the highest rates of sodium consumption, with fish sauce being one of the main sources. The aim of this study was to examine whether changes in the micro-environment factors can affect fish sauce consumption behavior in a university setting in Thailand. We implemented four interventions (with one control) in five canteens across a Thai university. The study design was a Latin square, where the five canteens were randomized over five weeks to implement four interventions plus a control. Our interventions included behavior-oriented, cognitive-oriented, and affective-oriented nudges aimed to reduce the amount of fish sauce people add to their noodles during lunchtime at the university canteens. Results indicate that a simple change in how fish sauce was served can reduce fish sauce consumption. Serving fish sauce in a bowl with a spoon reduced the amount of fish sauce used per noodle bowl by 0.25 grams, compared to the normal condition where fish sauce is served in a bottle. Using a specially-designed spoon with a hole induced a larger reduction of 0.58 grams of fish sauce used per bowl. The other two interventions, cognitive- and affective- oriented nudges, also showed reductions of fish sauce usage, but the differences were not statistically significant. The findings can be used for policy implementation to advocate the use of a smaller sized spoon and a bowl to serve fish sauce instead of a bottle to reduce sodium consumption among Thai people.

## Introduction

Non-communicable diseases (NCDs) are the leading cause of mortality and morbidity worldwide. One of the four major risk factors contributing to NCDs is unhealthy diet, where high consumption of sodium contributes to 4.1 million annual deaths [1]. Thailand is among the countries with the highest sodium consumption. According to Powles, Fahimi, Micha, et al., Thailand’s age-standardized estimated sodium intake for persons aged 20 and over in 1990 and 2010 were 5.23 and 5.31 grams/day, respectively [2]. This is more than the global sodium intake of 4.02 and 4.14 grams/day in 1990 and 2010, respectively [2], and substantially higher than the recommended intake by the World Health Organization at 2 grams/day [3].

The main source of sodium intake among Thais, like many other Southeast Asian countries, comes from condiments added to meals while cooking or at the table [4]. From a national representative survey by Thailand National Statistics Office in 2017, 55.8 percent of Thai people aged 6 years and older add condiments to meals at the table, with the highest rate among the 15–24 age group at 64.8 percent. The condiment that Thais add the most is fish sauce or soy sauce at 69 percent, followed by sugar at 40.5 percent, chili powder at 38 percent, pickled chili at 26.9 percent, and salt at 4.6 percent (multiple answers) [5]. Noodles are one of the most popular dishes among Thais. According to the Thai Food Consumption Survey conducted by Thailand National Health Exam Survey Office, 55.4 percent of Thai adults aged 15 years and over consume noodles every week, and 17.9 percent consume noodles almost every day [6]. A study conducted by Pavadhgul et al. among undergraduate students at Mahidol University in Thailand found that the main source of sodium was from individual-dish meals such as noodles and rice with stir-fried meat and holy basil leaves [7]. These dishes provide a mean sodium intake of 2,437.8 ± 1,376.9 mg/day. In addition, 64.7 percent of respondents reported adding condiments to noodles and rice with stir-fried meat and holy basil, with 89.1 percent of those who add condiments adding at least one teaspoon of fish sauce, increasing the sodium intake by 505.8 ± 506.5 mg/day [7].

### The health policy challenge

Thailand has campaigned to reduce sodium consumption for more than a decade. The first national strategy to reduce salt and sodium consumption in 2013 followed the World Health Organization (WHO) resolution adopted in May 2013 that set nine global NCD targets [8]. Reducing salt and sodium intake by 30 percent by the year 2025 was one of the nine global targets [8], and Thailand responded by adopting the national nine NCD targets at the 6th National Health Assembly in December 2013, where salt and sodium intake was set to be reduced by 30 percent [9]. Later, the 8th National Health Assembly in 2015 adopted a resolution addressing the policy on salt and sodium consumption reduction to address NCDs [10]. Subsequently, the Strategic Plan on Sodium and Salt Reduction in Thailand 2016–2025 was formulated under the Department of Disease Control, Ministry of Public Health [11].

For legislation and environmental reform, in 2016 the Ministry of Public Health announced two government Notifications of Mandatory Nutrition Labelling and a Guideline Daily Amount (GDA) including sodium content in 2016 and 2018 [12, 13]. The products that need to carry GDA nutrition fact labels include snacks, chocolates, bakery products, instant food products, frozen single-serve meals, drinks in sealed packages, tea, coffee, milk, yoghurt, dairy products, soy milk, and ice-cream [12, 13]. Note, however, that this mandatory nutrition labelling and GDA can only regulate processed manufactured foods, where street foods and ready-cooked foods served in canteens and food stalls are left unregulated.

Despite these ongoing efforts, the current sodium consumption in Thailand is still above the recommended levels [2, 3]. The interventions in this study are an attempt to provide a new way of approaching the problem. Our study adds to previous studies in the literature on sodium consumption in several ways. First, our study focuses on the behavior of adding condiments at the table, where most studies focus on food consumption rather than adding condiments. Second, this study examines the behavior of Thais which shares some similarity with those of other Asian countries such as the Philippines and Vietnam [4], where condiments are added at the table. Third, this is the first study applying behavior change approaches to address this public health and policy challenge.

### The behavior change solution

There is a growing literature providing empirical evidence on strategic changes in the environment (sometimes referred as nudges) and their influence on people’s behavior. To determine nudging interventions that influence and improve individuals’ health-related behaviors—including food and beverage consumption—recent studies emphasized the importance of micro-environment factors. The ideas are mostly based on a behavioral science approach which concerns the impacts of cognitive, emotional, and socio-cultural factors on the individuals’ decisions. Appropriate design and implementation of interventions to alter micro-environment elements were evidenced to affect those factors and ultimately individuals’ behaviors [14, 15]. The term choice architecture highlights the strategy of influencing choice of individuals or the population by setting or altering the contexts in which they make decisions.

The nudge approach to behavior change is derived from a dual process theory. According to psychological studies on how the human brain operates, two distinct systems for information processing and action control are identified—reflective and automatic [16]. By characteristics, the former system is more controlled, effortful, deductive, slow, and self-aware, while the latter is more uncontrolled, effortless, emotional, fast, and unconscious. The nudge approach emphasizes and covers mainly the influences on the automatic system of the human brain. For example, MINDSPACE, a well-known comprehensive framework for designing nudging interventions, provides a checklist of nine most robust influences on human behavior for use when designing interventions or policy making [17]. This approach has been applied in various case studies of policy making that aim to influence specific behaviors such as smoking and alcohol consumption, organ donation, diet and weight, physical activity, or food hygiene [18–20].

The majority of systematic reviews and meta-analyses suggest a small but positive effect of these strategic changes in altering behavior [21–24]. Since there can be many types of behavioral change interventions, analyses are typically categorized by the type of nudge. A meta-analysis of field experiments classified nudges for healthy dietary intake broadly into three groups [14]. These include: *cognitive*-oriented nudges such as descriptive and evaluative nutritional labelling or visibility enhancements; *affective*-oriented nudges such as hedonic or preference enhancements or healthy eating calls; and *behavior-*oriented nudges that influence selection, purchase, and food consumption behaviors by changing convenience (e.g., placement) or properties (e.g., size) of the objects or stimuli (e.g., see the Typology of Interventions in Proximal Physical Micro-Environments (TIPPME) [25]. Cadario and Chandon [22] conducted a meta-analysis of nudges and found that the type of nudge that showed the largest effect was behavior-oriented nudges (such as convenience or size enhancements), followed by affective-oriented nudges (such as hedonic enhancements and healthy eating calls). The least effective nudges were cognitive-oriented interventions (such as descriptive nutritional labeling, evaluative nutritional labeling, and visibility enhancements). Behavior-oriented nudges such as making unhealthy foods less convenient to consume, using less convenient serving utensils (tongs versus spoons), or offering smaller-sized utensils, have been shown to reduce the amount consumed [26, 27].

Studies also show large heterogeneity in effectiveness [24, 28], which implies those three types of nudges are all relevant to our public health challenge—reducing sodium consumption among adults eating in public venues. A recent systematic review of studies including cognitive nudges that are making specific information more salient or accessible (also known as priming) found a variation in the effectiveness of nudging since the populations and settings tested vary greatly [28]. Such nudges tend to be more effective in adults, as opposed to children; and, offsite eateries and onsite cafeterias are more effective settings compared to grocery stores [22]. The heterogeneity in effectiveness of nudges also depends on the type of behavior they are aiming to influence. For instance, do the nudges aim to promote healthy eating behaviors or to discourage unhealthy ones? From the meta-analysis, a 30 percent stronger effect size was found for reducing unhealthy eating than for increasing healthy eating [22]. With respect to behavior-oriented nudges, Rozin et al. [27] examined the effect of altering accessibility on food intake in a university cafeteria. The study found that aside from changing the order and position of foods in the salad bar, changing the serving utensil from spoons to tongs reduced intake of unhealthy foods in the salad bar in the range of 8–16 percent.

Empirical evidence shows that food consumption can be altered by making strategic changes in the environment. However, these studies were conducted predominantly in the Western context, particularly in the US. Food cultures in Asia differ vastly from the West, and even among Asian countries there are enormous variations. Therefore, this study will explore nudge interventions, including cognitive-oriented, behavior-oriented, and affective-oriented approaches, in reducing the amount of fish sauce consumers add at the table.

## Methods

The main objective of this study was to assess the potential impacts of interventions that adjust micro-environmental elements on the quantity of fish sauce added to noodles by consumers, in the context of Thai university canteens.

### Study sites

Five canteens of Mahidol University, Salaya Campus, Nakhon Pathom, Thailand were selected as the study sites from a total of six canteens. We included all canteens available on campus (apart from one canteen which was designated for another study). The criteria for selecting the canteens were that they must have at least one noodle shop and the shop owner must provide consent and cooperation to participate in the study. All canteens received consent from the department or faculty responsible for each canteen. We approached each noodle shop to explain our research study and to ask for their consent. All noodle shops in all five canteens accepted to participate in our study. In total, there were 11 noodle shops from five canteens.

### Interventions

In the control scenario, fish sauce was served in a 750 ml bottle, placed in front of each noodle shop. After the consumers buy their noodles, they can choose if they want to add any condiments to their desired taste. Based on the concepts of healthy eating nudges—including cognitive-oriented, affective-oriented, and behavior-oriented nudges [14], this study tested the following four interventions:

1. A behavior-oriented nudge by changing the convenience of objects. The fish sauce serving container was changed to a bowl (with 500 g of fish sauce) with a “regular spoon” (that can extract 15 g of fish sauce). Using a spoon to extract fish sauce from a bowl requires more effort than extracting fish sauce directly from a bottle, and therefore may reduce the quantity of fish sauce added to the noodles.
2. A behavior-oriented nudge by changing the convenience and property of objects. Similar to the first intervention, the fish sauce serving container was changed to a bowl (with 500 g of fish sauce). But instead of using a regular spoon, a special spoon designed by the Thai Health Promotion Foundation to reduce sodium consumption among Thai people was used. The special spoon, named the “less spoon”, has a hole in the middle and can extract 3.3 g of fish sauce at a time, or approximately two thirds of a teaspoon. Displayed near the bowl of fish sauce was an A4-sized information sheet introducing the “less spoon” with the short message: “Do not add more than 2/3 teaspoons of fish sauce per meal”. This intervention was expected to make adding fish sauce even less convenient while prompting the noodle buyers not to add more than the recommended amount of fish sauce.
3. A cognitive-oriented nudge by providing information about health and sodium consumption. In this intervention, fish sauce was served in a bottle, just like in the control scenario, but an A4-sized information sheet was also displayed near the fish sauce bottle. The sheet provided information regarding the amount of sodium already in noodles and the recommended sodium consumption per meal. The information sheet also showed a picture of a smartwatch with a healthy heart, a 120/80 blood pressure reading, a positive reinforcement message “GREAT JOB”, and a short message reading: “Less salty, reduces disease, weight, and bloating”. The display of the information sheet was expected to enhance the saliency of health and healthy living with better cognitive knowledge on the amount of sodium in the diet, and awareness about risks of excess sodium intake.
4. An affective-oriented nudge by providing information about health and sodium consumption with an emotional stimulus. In this intervention, fish sauce was served in a bottle, just like in the control scenario, but an A4-sized information sheet with an affective-provoking picture was also displayed nearby the fish sauce bottle. The sheet provided information regarding the amount of sodium already in noodles and the recommended sodium consumption per meal. In addition, there was a picture of a girl making a disgusted face with a short message saying “too salty”. Evidence shows that the human amygdala exerts some of its emotion regulation functions when the observer is exposed to (but not necessarily aware of) the triggering emotional stimulus such as facial expressions [29].
5. No intervention as the control—i.e., standard noodle shop practice of serving fish sauce in a bottle.

**Fig 1:**
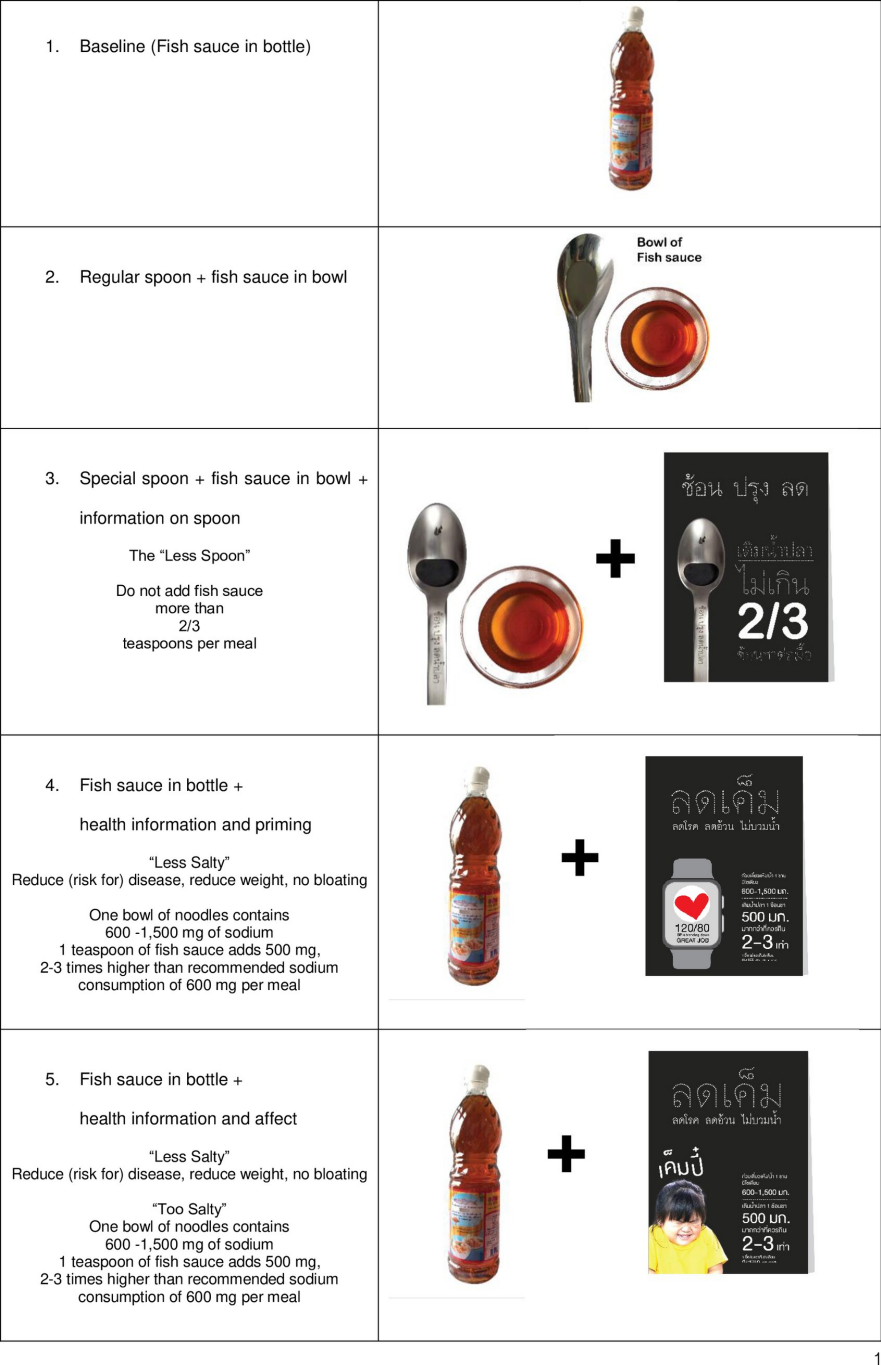
Pictures showing interventions used in the experiment.

Corresponding pictures for control and interventions 1–5 are shown in Fig 1.

### Study design

The unit of analysis in this experiment is canteen, not an individual noodle shop. This is due to the fact that the contexts and conditions of the shops located in the same canteen, in terms of characteristics of regular consumers and other physical settings and environments, are very similar; and, it is difficult to prevent carryover effects of the intervention being experimented with a noodle shop to the others in the canteen. With this condition, the number of canteens was limited to five. Due to the number of available canteens, the time limitation, and financial budget available, a Latin square design was applied in this experiment for the primary analysis. The implementation plan of interventions (including four randomized interventions and no intervention for control) in five canteens (randomized Canteens A-E) within a five-week period (Weeks 1–5, five days per week) is presented in Table 1.

**Table 1:** Latin square design of interventions across weeks and canteens.

	Week 1	Week 2	Week 3	Week 4	Week 5
	9–13 September 2019	16–20 September 2019	23–27 September 2019	7–11 October 2019	4–8 November 2019
Canteen A	Control	Info + priming	Special spoon	Info + affect	Regular spoon
Canteen B	Regular spoon	Control	Info + priming	Special spoon	Info + affect
Canteen C	Info + affect	Regular spoon	Control	Info + priming	Special spoon
Canteen D	Special spoon	Info + affect	Regular spoon	Control	Info + priming
Canteen E	Info + priming	Special spoon	Info + affect	Regular spoon	Control

This Latin square study design included the intervention effect and two nuisance effects, namely canteen effect and week effect, since no inter-week carryover effects were expected. Canteens were randomized within the Latin square design. Data was collected on weekdays during the peak lunch time (11:30 am–1:30 pm) over a period of five weeks during September to November 2019. Due to special events in some weeks during the data collection period (i.e., graduation week, mid-term examination week, and several national holidays in October), data collection was halted for a total of five weeks (not all consecutively) since the number of students on campus differed from the other weeks. During the weeks when data collection was halted, the most recent intervention for each canteen still continued; there were no suspensions of interventions during the data collection break. One daily observation was not collected during the study for canteen B, due to the noodle shops within the canteen being closed unannounced. This was an unexpected closure, and we treated this observation as missing in the analysis. With five canteens over the experimental period of five weeks (weekday only), the total number of observations for this study was 124.

Three data collection teams were created and trained about the experimental protocols and report forms. Responsible for 3–4 shops a day, each team comprised 3–4 data collectors (one per shop, preparing intervention settings and collecting data) and one team supervisor (coordinating with the main research team through the field coordinator, supervising the data collectors, and inspecting the quality of data collection). Their main responsibilities included setting up the intervention, monitoring the set up (e.g. whether fish sauce needed to be refilled, or whether the setup had been moved), observing consumers’ behaviors, and measuring the amount of fish sauce used each day. The main research team rotated among the canteens to observe the data collection process.

Each day before the start of data collection, the data collection team prepared the fish sauce according to the implementation plan for each canteen and set up the intervention in front of each noodle shop. The fish sauce used was the same brand for all the noodle shops throughout the experimental period for consistency, the bowls used were also the same in all sites, and the amount of the fish sauce offered in a bowl was measured to be 500 grams at the start of the collection period. If the fish sauce depleted to less than half the container during the experimental period, the researcher replaced the fish sauce in a new serving container and weighed and recorded the remaining fish sauce in the old container.

The data recorded each day during the experimental period came from direct observation, which included the number of noodle bowls sold and the number of noodle bowls to which fish sauce was added (by the consumer). Data collectors observed consumers’ behaviors from a distance using two tally counters. One counter was for counting the total number of noodle bowls sold, and another was for counting the bowls to which the consumers added fish sauce. The team weighed the fish sauce in its container on a scale twice each day; before and after the experimental period. The total quantity of fish sauce used per day per noodle shop (in grams) came from subtracting the weight of the remaining fish sauce in the serving container at the end of the collection period from the weight at the start of the collection period in each day. Since the difference in the weight could only come from the reduction in fish sauce, there was no need to take into account the weight of the empty containers. Data collectors were rotated every day to a different noodle shop to avoid individual bias and any potential data collector effect that might occur with a specific shop.

### Data analysis

A single outcome was assessed in this study: average fish sauce usage. However, three different measurements of this outcome were analyzed: (1) the amount of fish sauce added (in grams) divided by the total number of bowls sold per day, which is the primary measurement of the outcome; (2) the amount of fish sauce added as a proportion of the total number of bowls to which fish sauce was added per day; and (3) the percentage of bowls to which fish sauce was added, defined as a proportion over the total number of fish bowls sold per day. These outcome measurements are expected to be correlated, since they relate to alternative measurements of the same outcome event. Therefore, each analysis is treated as an alternative perspective of the same underlying outcome, broadly defined as the extent of the use of fish sauce following intervention or control/no intervention. It is important to note that our measures do not reflect the amount of fish sauce actually consumed, but rather how much fish sauce was added to the noodles.

Noodle bowl sizes were very similar among canteens, so no further adjustment for bowl size was needed. Also, no major events were reported during the weeks of data collection that would have added new sources of variability to the data.

The data analysis included descriptive and inferential statistics. The descriptive statistics showed the mean and standard deviation of each outcome across interventions, as well as canteens, weeks, and days of the week.

As for the primary analysis model resulting from the Latin square design, it can be defined as follows across the different outcome measurements:

$$Outcome_{i,j,k}=\alpha +Intervention_{i}+Canteen_{j}+Week_{k}+Error_{i,j,k}$$

The reference categories used to calculate intra-block effects include: No Intervention/Control; Canteen B; and Week 1.

A secondary analysis was performed by replacing the week effect with the day-of-the-week effect (which is feasible under the current design of five time blocks with daily measurements), also controlling for canteen effects. This secondary analysis accounts for a potential alternative seasonality effect, by accounting for weekday effects instead of weekly effects. This results in the following secondary model:

$$Outcome_{i,j,k}=\alpha +Intervention_{i}+Canteen_{j}+Weekday_{k}+Error_{i,j,k}$$

The reference categories used to calculate intra-block effects include: No Intervention/Control; Canteen B; and Monday. The analysis included parameter estimates, standard errors, t-value, p-value, and the 95% confidence interval. The significance level in this study was set to 5%. All statistical analyses were performed using R version 3.6.1.

### Ethical approval

Research ethical clearance was obtained prior to the study from the Institute for Population and Social Research Institutional Review Board (IPSR-IRB), Mahidol University, with the certificate of approval COA. No. 2019/08-347. All department or faculty responsible for each canteen provided consent prior to the study. In addition, each noodle shop was approached to explain the research study and to ask for their written consent. As for collecting data on fish sauce consumption behaviors during the study, we only observed from a distance whether each person added fish sauce to their bowl, and therefore did not approach individual consumers for consent.

## Results

Table 2 shows summary statistics across the three outcome measurements by intervention, canteen, and week for the primary analysis, as well as the secondary analysis in which week is replaced by weekday. When measured as the amount of fish sauce used (in grams) out of either the number of bowls sold or the number of bowls to which fish sauce was added, there is a noticeably smaller observed mean and standard deviation for the intervention containing the special spoon and information, relative to the control/no intervention and all other interventions. Tables 3–5 contain analysis results for each of the three outcome measurements, respectively. Results include model parameter estimates and corresponding standard errors, t-statistics, p-values, and 95% confidence intervals for each intervention, canteen, and week.

**Table 2:** Descriptive statistics for the outcomes across interventions, canteens, and week (for the primary analysis) or weekday (for the secondary analysis).

	Fish sauce used (grams)/Bowls sold per day		Fish sauce used (grams)/Bowls to which fish sauce was added per day		% Bowls to which fish sauce was added/Bowls sold per day	
	Mean (grams/bowl)	SD (grams/bowl)	Mean (grams/bowl)	SD (grams/bowl)	Mean (%)	SD (%)
Intervention						
Control/No intervention	1.38	0.74	5.91	2.44	23.05	7.55
Regular spoon	1.13	0.34	4.71	1.39	24.84	7.00
Special spoon + information	0.80	0.31	3.27	0.97	24.61	8.05
Information + priming picture	1.30	0.46	5.34	1.76	24.57	6.28
Information + affect picture	1.18	0.37	5.31	1.89	23.05	6.66
Canteen						
A	0.94	0.22	4.49	1.07	21.07	2.87
B	1.35	0.24	4.56	0.98	30.05	3.90
C	0.93	0.55	4.65	1.80	19.51	5.07
D	1.01	0.42	4.17	1.90	25.17	9.24
E	1.57	0.60	6.63	2.55	24.58	7.30
Week						
1	1.27	0.48	5.00	2.29	27.18	8.40
2	1.15	0.54	4.66	2.04	24.60	6.92
3	1.21	0.68	4.94	2.25	24.40	6.71
4	1.03	0.33	4.59	1.07	22.50	5.50
5	1.11	0.40	5.30	1.88	21.41	6.53
Weekday						
Monday	1.08	0.47	4.74	1.88	22.76	6.22
Tuesday	1.19	0.60	5.25	2.33	22.93	6.24
Wednesday	1.16	0.46	4.49	1.54	26.17	7.71
Thursday	1.18	0.46	4.63	1.57	25.88	7.63
Friday	1.17	0.54	5.41	2.28	22.32	6.93

**Table 3:** Model results for the outcome measurement: Amount of fish sauce used (grams) per bowl sold per day.

	Estimate	Standard Error	t-value	p-value	95% Confidence Interval (CI)
Reference mean^a^	1.6849	0.1295	13.011	<0.0001	(1.4283,1.9416)
Intervention^a^					
Regular spoon	-0.2509	0.1124	-2.232	0.0276	(-0.4736,-0.0282)
Special spoon + information	-0.5768	0.1124	-5.131	<0.0001	(-0.7995,-0.3540)
Information + priming picture	-0.0844	0.1124	-0.751	0.4541	(-0.3072,0.1383)
Information + affect picture	-0.1962	0.1124	-1.745	0.0837	(-0.4189,0.0266)
Canteen^a^					
A	-0.4152	0.1124	-3.694	0.0003	(-0.6379,-0.1925)
C	-0.4246	0.1124	-3.778	0.0003	(-0.6474,-0.2019)
D	-0.3445	0.1124	-3.065	0.0027	(-0.5672,-0.1217)
E	0.2171	0.1124	1.932	0.0560	(-0.0056,0.5498)
Week^a^					
2	-0.1191	0.1112	-1.072	0.2861	(-0.3394,0.1011)
3	-0.0579	0.1112	-0.521	0.6034	(-0.2782,0.1624)
4	-0.2262	0.1124	-2.012	0.0466	(-0.4489,-0.0034)
5	-0.1558	0.1112	-1.401	0.1639	(-0.3761,0.0645)
Model statistics					
R-squared = 0.4467	Adj-R-squared = 0.3869		F(12,111) = 7.469		p-value<0.0001

^a^Reference categories for each variable used to calculate intra-block effects include: No Intervention/Control; Canteen B; and Week 1

**Table 4:** Model results for the outcome measurement: Amount of fish sauce used (grams) per bowl to which fish sauce was added per day.

	Estimate	Standard Error	t-value	p-value	95% Confidence Interval (CI)
Reference mean^a^	5.6667	0.5064	11.190	<0.0001	(4.6632,6.6701)
Intervention^a^					
Regular spoon	-1.1721	0.4395	-2.667	0.0088	(-2.0430,-0.3012)
Special spoon + information	-2.6120	0.4395	-5.943	<0.0001	(-3.4829,-1.7411)
Information + priming picture	-0.5421	0.4395	-1.233	0.2200	(-1.4130,0.3288)
Information + affect picture	-0.5719	0.4395	-1.301	0.1959	(-1.4428,0.2991)
Canteen^a^					
A	-0.0979	0.4395	-0.223	0.8241	(-.9688,0.7730)
C	0.0583	0.4395	0.133	0.8947	(-0.8126,0.9292)
D	-0.4174	0.4395	-0.950	0.3443	(-1.2883,0.4535)
E	2.0439	0.4395	4.650	<0.0001	(1.1728,2.9146)
Week^a^					
2	-0.3414	0.4347	-0.785	0.4339	(-1.2028,0.5199)
3	-0.0653	0.4347	-0.150	0.8809	(-0.9266,0.7960)
4	-0.3863	0.4395	-0.879	0.3814	(-1.2572,0.4847)
5	0.2958	0.4347	0.681	0.4976	(-0.5655,1.1572)
Model statistics					
R-squared = 0.4383	Adj-R-squared = 0.3776		F(12,111) = 7.217		p-value<0.0001

^a^Reference categories for each variable used to calculate intra-block effects include: No Intervention/Control; Canteen B; and Week 1

**Table 5:** Model results for the outcome measurement: % Bowls to which fish sauce was added per bowl sold per day.

	Estimate	Standard Error	t-value	p-value	95% Confidence Interval (CI)
Reference mean^a^	0.3225	0.0197	16.401	<0.0001	(0.2835,0.3615)
Intervention^a^					
Regular spoon	0.0160	0.0171	0.939	0.3499	(-0.0178,0.0498)
Special spoon + information	0.0378	0.0171	0.807	0.4212	(-0.0200,0.0476)
Information + priming picture	0.0133	0.0171	0.781	0.4368	(-0.0205,0.0471)
Information + affect picture	-0.0019	0.0171	-0.109	0.9137	(-0.0357,0.0320)
Canteen^a^					
A	-0.0889	0.0171	-5.209	<0.0001	(-0.1227,-0.0551)
C	-0.1045	0.0171	-6.121	<0.0001	(-0.1383,-0.0787)
D	-0.0479	0.0171	-2.805	0.0059	(-0.0817,-0.0141)
E	-0.0538	0.0171	-3.151	0.0021	(-0.0876,-0.0200)
Week^a^					
2	-0.0258	0.0169	-1.526	0.1299	(-0.0592,0.0077)
3	-0.0278	0.0169	-1.645	0.1028	(-0.0612,0.0057)
4	-0.0446	0.0171	-2.615	0.0102	(-0.0784,-0.0108)
5	-0.0576	0.0169	-3.414	0.0009	(-0.0911,-0.0242)
Model statistics					
R-squared = 0.3554	Adj-R-squared = 0.2857		F(12,111) = 5.100		p-value<0.0001

^a^Reference categories for each variable used to calculate intra-block effects include: No Intervention/Control; Canteen B; and Week 1

Our findings show that our interventions were able to reduce the total quantity of fish sauce added. In the control scenario where fish sauce was offered in a bottle, people on average added 1.38 grams of fish sauce per noodle bowl sold. Changing from a bottle to a bowl with a regular spoon, the amount of fish sauce added decreased to 1.13 grams, which is equivalent to a reduction of 21.6 milligrams of sodium (calculated based on the nutrition label on the fish sauce bottle). The quantity of fish sauce added was further reduced to 0.80 grams once we replaced the regular spoon with a special spoon. This is equivalent to a reduction of 51.3 milligrams of sodium per all bowls of noodles. The other two interventions with priming and affect signs also reduced the amount of fish sauce added, but the effects were small and not statistically significant.

Upon closer inspection of the behavior, we found that the interventions were not effective in changing people’s decisions whether to add fish sauce, but rather the quantity of fish sauce added among those who added fish sauce. In the control scenario, an average of about 23 percent of people across all five canteens added fish sauce to their bowl of noodles. With the interventions in place, the proportion of people who added fish sauce varied between approximately 23–25 percent, which was not statistically different from the control (Table 2).

Among those who added fish sauce, the reduction in the quantity of fish sauce used was further pronounced with interventions. Considering only those who added fish sauce, on average 5.91 grams of fish sauce was added in the control scenario. With fish sauce offered in a bowl and a regular spoon, the amount reduced to 4.71 grams, or a 101.7 milligrams reduction of sodium. The intervention with a bowl and a special spoon resulted in the biggest reduction of fish sauce added among those who used fish sauce, decreasing to 3.27 grams, or a reduction of 231.3 milligrams of sodium.

Statistically significant differences were found when comparing two of the interventions to the control/no intervention for outcome measurements pertaining to actual amounts of fish sauce usage (Tables 3 and 4). The interventions using the (1) regular spoon and (2) special spoon combined with information offer statistically significant improvements in amounts of fish sauce used compared to the control/no intervention. Use of a regular spoon induced an estimated reduction of 0.2509 grams of fish sauce used per bowl sold per day (p = 0.0276; 95% CI -0.4736,-0.0282), or a reduction of 1.1721 grams of fish sauce used per bowl to which fish sauce was added per day (p = 0.0088; 95% CI -2.0430,-0.3012) compared with the control of using the standard fish sauce bottle. Use of the special spoon combined with information induced a larger reduction, an estimated reduction of 0.5768 grams of fish sauce used per bowl sold per day (p<0.0001; 95% CI -0.7995,-0.3540), or a reduction of 2.6120 grams of fish sauce used per bowl to which fish sauce was added per day (p<0.0001; 95% CI -3.4829,-1.7411) compared to the control of the standard bottle of fish sauce used by canteens.

The other two interventions (3) cognitive nudging and (4) affective nudging also show estimated reductions in these two outcome measurements of fish sauce usage, though differences are statistically non-significant ((3) p = 0.4541; 95% CI -0.3072,0.1383 and p = 0.2200; 95% CI -1.4130,0.3288; and (4) p = 0.0837; 95% CI -0.4189,0.0266 and p = 0.1959; 95% CI -1.4428,0.2991, respectively—see Tables 3 and 4). There are no significant differences across interventions compared to the control/no intervention (p-values range from 0.3499 to 0.9137) for the binary outcome measurement representing fish sauce usage—i.e., the percentage of bowls to which fish sauce was added per bowls sold per day.

Canteen differences appear significant across multiple outcome measurements. For the outcome measurement of the amount of fish sauce used (grams) per bowl sold per day (Table 3), canteens A, C, and D saw significant reductions (in grams) of 0.4152 (p = 0.0003; 95% CI -0.6379,-0.1925), 0.4246 (p = 0.0003; 95% CI -0.6474,-0.2019), and 0.3445 (p = 0.0027; 95% CI -0.5672,-0.1217), respectively, in fish sauce usage compared to the reference canteen B. Whereas, canteen E saw a non-significant (p = 0.0560; 95% CI -0.0056,0.5498) increase of 0.2171 grams. For the outcome measurement of the amount of fish sauce used (grams) per bowl to which fish sauce was added per day (Table 4), the customers frequenting canteen E used a significantly larger (p<0.0001; 95% CI 1.1728,2.9146) amount of fish sauce (2.0439 grams more) compared to the reference canteen B. All other canteens had non-significant reductions or increases (p>0.3443). Though, all canteens saw significant reductions, with p-values ranging from <0.0001 to 0.0059, of 4.79% (D) to 10.45% (C) in the percentage of bowls to which fish sauce was added per bowl sold per day compared to the reference canteen B (Table 5).

Weekly effects were mostly negligible except for Week 4 for fish sauce used per bowl sold (Table 3; p = 0.0466; 95% CI -0.4489,-0.0034) and Weeks 4 and 5 for the percentage of bowls with added fish sauce (Table 5; p = 0.0102; 95% CI -7.84%,-1.08% and p = 0.0009; 95% CI -9.11%,-2.42%, respectively).

The last row in each of Tables 3–5 presents overall model statistics across outcome measurements, including r-squared, adjusted r-squared, F-statistic, and p-value. R-squared values indicate that the explained variability ranges from approximately 36% to 45% across these models, with p<0.0001 in all cases.

Results of the secondary analysis, in which the week effect was replaced by a day-of-the-week effect in the model, are reported in S2–S5 Tables. Similar to the primary analysis results, the regular spoon and special spoon with information interventions were associated with statistically significant reductions in amount of fish sauce used per bowl sold and per bowl to which fish sauce was added. The same canteens shown to have significant associations with the outcome measurements in the primary analysis were also the case in this secondary analysis. No weekday effects were observed across the outcome measurements, with p-values ranging from 0.0506 to 0.9273. Similar to primary analysis results, the explained variability ranges from approximately 33% to 46% across these models, again with p<0.0001 in all cases.

## Discussion

Thailand has set a target to reduce sodium consumption by 30% by the year 2025 following the global target set by the World Health Organization. However, sodium consumption in Thailand remains high despite the efforts made by all stakeholders. Adding fish sauce or soy sauce to meals at the table increases sodium intake by 350–500 mg per teaspoon. This study used nudge theory to develop interventions to change the behavior of adding fish sauce to noodle bowls by customers in the canteen at Mahidol University in Thailand.

This study found that using a behavior-oriented nudge by changing the fish sauce serving container from a bottle to a bowl with a spoon can reduce the amount of fish sauce added by 0.2509 grams per bowl sold per day (p = 0.0276; 95% CI -0.4736,-0.0282), or a reduction of 1.1721 grams per bowl to which fish sauce was added per day (p = 0.0088; 95% CI -2.0430,-0.3012). The use of the special spoon combined with information induced a larger reduction, an estimated reduction of 0.5768 grams of fish sauce used per bowl sold per day (p<0.0001; 95% CI -0.7995,-0.3540), or a reduction of 2.6120 grams of fish sauce used per bowl to which fish sauce was added per day (p<0.0001; 95% CI -3.4829,-1.7411), compared to the control of the standard bottle of fish sauce used by canteens. The reduction in fish sauce added helps reduce the sodium intake by 26.6 and 61.1 g per bowl sold per day using a regular spoon and special spoon, respectively, and 124.2 g and 276.9 g per bowl to which fish sauce was added using a regular spoon and special spoon, respectively. However, none of the nudge interventions resulted in changing the behavior of adding fish sauce to consumers’ noodle bowls. Our findings show no significant differences across interventions compared to the control/no intervention (p-values range from 0.3499 to 0.9137) for the binary outcome measurement representing fish sauce usage—i.e., the percentage of bowls to which fish sauce was added per bowl sold per day. This means that the current interventions do not affect consumers’ decision to add fish sauce. To discourage fish sauce usage, other types of interventions must be explored. One option is to change the availability or the convenience of getting fish sauce, such as by placing the condiment station further away from the noodle shop [30].

Based on the Nudge Theory, changing the fish sauce container from a bottle to a bowl with a spoon is a behavior-oriented nudge. Using a spoon to extract fish sauce from a bowl takes more time and effort for the customers to get the same amount of fish sauce from a bowl than from a bottle. As a result, customers reduce the amount of fish sauce added even without intending to do so. The use of a special spoon with a hole further prompts customers to better gauge the amount of fish sauce they are adding to their noodle bowl compared to pouring fish sauce straight from a bottle. In this intervention, the special spoon was introduced alongside a sign that read “do not add more than 2/3 teaspoons of fish sauce per meal”. The special spoon cues customers to visually see and achieve the recommended amount. Our findings align with previous studies showing that behavior-oriented nudges are most effective in changing consumption behavior, such as making unhealthy foods less convenient to consume by using less convenient serving utensils (tongs versus spoons) or offering smaller-sized utensils [22, 26, 27].

The other two interventions with cognitive and affective nudges were not as effective in reducing the amount of fish sauce added. The mechanisms intended behind these interventions were to change behavior in an automatic way. The cognitive-oriented nudge attempted to give a subconscious cue (priming) to think about one’s health, while the affect sign was an affective-oriented nudge to trigger a feeling of “too salty” that would deter adding fish sauce. In our study, these two interventions were not effective. Possible reasons may be because university students may still be too young to be concerned about health, and therefore health primes would not trigger a subconscious cue to add less fish sauce. For priming to work, the sign must trigger their automatic and intuitive behavior through an existing personal long-term goal in health. Previous studies of priming nudges on eating behavior have shown mixed results [28, 31]. Among those with positive outcomes, the effect is limited to certain groups of populations, particularly when the priming cues align with individuals’ goals. For instance, health primes were more effective in reducing consumption of those on a diet than those who were not [32–35]. Similar arguments can be made for the affect sign. People who typically add fish sauce in their noodles are more likely to prefer flavorful foods and may not be concerned about their food being “too salty”. Therefore, the visual cue to trigger a feeling of food being “too salty” does not activate any existing goals to eat less salty foods.

External factors may also contribute to the ineffectiveness of cognitive (priming) and affect interventions. The noodle shops were small in size, and we were limited as to where we could place the information sheet. For instance, we were not always able to place the sheet at eye level or close enough to the fish sauce bottle. Moreover, there were typically many customers occupying the front of the shop during busy hours, crowding out the sign. In addition, many shops typically have other signs already in place, e.g. menus, announcements from the university, and other health-related information sheets, making our sign not as noticeable as it should be. Given these environmental settings, many people may have missed the information sheet or may not have had time to take a look. Many studies support this observation, as it has been shown that real-life settings often have many distractions that can reduce the effectiveness of interventions compared to interventions implemented in laboratory settings [36–38].

Overall, our results align with Cadario and Chandon’s [22] meta-analysis of nudges, which found that the type of nudge showing the largest effect was behavior-oriented nudges followed by affective-oriented nudges, with the least effective nudges being cognitive-oriented interventions.

It was interesting to find significant canteen differences for some of the outcome measurements. This could be an indication of either differences in the noodles provided (e.g., some canteens may provide noodles that are tastier with larger amounts of fish sauce already added), differences in the subpopulations being served by those canteens (e.g., faculty may prefer to use a particular canteen; there could be gender or age differences in the subpopulations that may explain canteen-specific differences), or other canteen-specific factors not captured by the model. One difference in subpopulations that can be observed from different canteens is that canteen C has more foreign students and faculty who frequent it compared to other canteens. The fish sauce adding behavior is more common among Thais and may explain why canteen C had the lowest percentage of bowls to which fish sauce was added and lowest amount of fish sauce used per bowls sold per day. But when compared with fish sauce used per bowls to which fish sauce was added, there was no difference.

Non-significant results or minimal differences by week or weekday blocking factors were expected, since menus offered were not changed throughout the week, and no significant intra- or inter-week differences were expected a priori in terms of the consumer population.

In terms of the limitations of the study, the outcome measurement reflected only how much fish sauce was added to the noodle bowls, not how much fish sauce was actually consumed, which the latter would be a more accurate measure of the potential impact on one’s health. As for the Latin study design, it must be taken into account that consumers may visit more than one canteen over the course of the experimental period. This means that any given consumer may be exposed to several interventions within the same week, which may have an impact on the outcomes.

In summary, this study supports the current literature that changes in the micro-environment can be effective in altering behavior. In our case, behavior-oriented nudges that encourage physical capability through the use of a fish sauce bowl and spoon was shown to be the most effective as they empowered people to better gauge the amount of fish sauce they were adding to their noodles, while also making it less convenient. Policy to reduce sodium intake in the Thai population can build on these behavioral insights by developing policy guidelines changing how fish sauce is served, e.g. from a bottle to a bowl with a spoon. The size of the spoon matters in terms of the amount of fish sauce added; the smaller the spoon, the less fish sauce is added. Using a regular spoon, but smaller in size, can be as effective as the special spoon, but more practical and cost-effective as there are no additional production costs. From the study, it is also recommended that there should be a lid to cover the bowl of fish sauce for sanitary reasons. The behavior-oriented nudge to reduce sodium intake in the population by changing the serving container to be less convenient for customers can be applied to other settings in Southeast Asian countries where adding condiments at the table is widely practiced. As for priming and affective-oriented nudges, further studies are required to better tailor messages that align more with the target population’s goals. For consumers to stop adding fish sauce altogether, further studies are needed to explore other types of interventions, especially the availability and proximity of fish sauce.

## Supporting information

S1 Table Intervention, number of bowls sold, grams of fish sauce used, and number of bowls in which fish sauce was added by week. (DOCX)

S2 Table Model results for the outcome measurement: Amount of fish sauce used (grams) per bowl sold per day for the secondary analysis. (DOCX)

S3 Table Model results for the outcome measurement: Amount of fish sauce used (grams) per bowl to which fish sauce was added per day for the secondary analysis. (DOCX)

S4 Table Model results for the outcome measurement: % Bowls to which fish sauce was added per bowl sold per day for the secondary analysis. (DOCX)

S5 Table Overall model statistics across outcome measurements for the secondary analysis. (DOCX)

S1 Dataset (ZIP)
