# Supplementary material for: Nudge interventions to reduce fish sauce consumption in Thailand
Source: PLoS One. 2020 Sep 8;15(9):e0238642. doi: 10.1371/journal.pone.0238642 (PMC7478907; doi:10.1371/journal.pone.0238642)
Supplement: S1 Table — (DOCX) [file pone.0238642.s001.docx]

|  | | Week 1  9–13 September 2019 | Week 2  16–20 September 2019 | Week 3  23–27 September 2019 | Week 4  7–11 October 2019 | Week 5  4–8 November 2019 |
| --- | --- | --- | --- | --- | --- | --- |
| Canteen A | Intervention | Baseline | Info + priming | Special spoon | Info + affect | Regular spoon |
|  | Number of bowls sold | 1,080 | 1,066 | 1,049 | 1,060 | 1,058 |
|  | Fish sauce used (grams) | 1,459 | 1,633 | 1,207 | 1,507 | 1,292 |
|  | Number of bowls to which fish sauce was added | 285 | 319 | 305 | 317 | 355 |
| Canteen B | Intervention | Regular spoon | Baseline | Info + priming | Special spoon | Info + affect |
|  | Number of bowls sold | 124 | 130 | 227 | 176 | 131 |
|  | Fish sauce used (grams) | 140 | 114 | 250 | 149 | 156 |
|  | Number of bowls to which fish sauce was added | 35 | 20 | 60 | 54 | 34 |
| Canteen C | Intervention | Info + affect | Regular spoon | Baseline | Info + priming | Special spoon |
|  | Number of bowls sold | 819 | 754 | 693 | 774 | 781 |
|  | Fish sauce used (grams) | 699 | 598 | 935 | 655 | 338 |
|  | Number of bowls to which fish sauce was added | 136 | 145 | 163 | 142 | 136 |
| Canteen D | Intervention | Special spoon | Info + affect | Regular spoon | Baseline | Info + priming |
|  | Number of bowls sold | 1,070 | 1,130 | 1,241 | 1,266 | 1,099 |
|  | Fish sauce used (grams) | 691 | 1,148 | 1,140 | 1,195 | 1,155 |
|  | Number of bowls to which fish sauce was added | 242 | 217 | 240 | 282 | 231 |
| Canteen E | Intervention | Info + priming | Special spoon | Info + affect | Regular spoon | Baseline |
|  | Number of bowls sold | 259 | 277 | 298 | 274 | 239 |
|  | Fish sauce used (grams) | 499 | 245 | 431 | 438 | 408 |
|  | Number of bowls to which fish sauce was added | 66 | 65 | 74 | 70 | 56 |
