# Supplementary material for: Nudge interventions to reduce fish sauce consumption in Thailand
Source: PLoS One. 2020 Sep 8;15(9):e0238642. doi: 10.1371/journal.pone.0238642 (PMC7478907; doi:10.1371/journal.pone.0238642)
Supplement: S2 Table — (DOCX) [file pone.0238642.s002.docx]

|  | **Estimate** | **Standard Error** | **t-value** | **p-value** | **95% Confidence Interval (CI)** |
| --- | --- | --- | --- | --- | --- |
| Reference mean | 1.5018 | 0.1315 | 11.424 | <0.0001 | (1.2413, 1.7624) |
| Intervention^a^ | | | | | |
| Regular spoon | -0.2571 | 0.1141 | -2.253 | 0.0262 | (-0.4831, -0.0309) |
| Special spoon + information | -0.5829 | 0.1141 | -5.109 | <0.0001 | (-0.8090, -0.3568) |
| Information + priming picture | -0.0906 | 0.1141 | -0.794 | 0.4290 | (-0.3167, 0.1355) |
| Information + affect picture | -0.2023 | 0.1141 | -1.773 | 0.0789 | (-0.4284, 0.0238) |
| Canteen^a^ | | | | | |
| A | -0.4213 | 0.1141 | -3.692 | 0.0003 | (-0.6474, -0.1952) |
| C | -0.4308 | 0.1141 | -3.775 | 0.0003 | (-0.6569, -0.2047) |
| D | -0.3506 | 0.1141 | -3.073 | 0.0027 | (-0.5767, -0.1245) |
| E | 0.2110 | 0.1141 | 1.849 | 0.0671 | (-0.0151, 0.4371) |
| Weekday^a^ | | | | | |
| Tuesday | 0.1131 | 0.1129 | 1.003 | 0.3183 | (-0.1105, 0.3368) |
| Wednesday | 0.0836 | 0.1129 | 0.741 | 0.4604 | (-0.1400, 0.3072) |
| Thursday | 0.1045 | 0.1129 | 0.926 | 0.3563 | (-0.1191, 0.3281) |
| Friday | 0.1104 | 0.1129 | 0.968 | 0.3353 | (-0.1157, 0.3365) |

^a^Reference categories for each variable used to calculate intra-block effects include: No Intervention/Control; Canteen B; and Monday
