# Supplementary material for: Nudge interventions to reduce fish sauce consumption in Thailand
Source: PLoS One. 2020 Sep 8;15(9):e0238642. doi: 10.1371/journal.pone.0238642 (PMC7478907; doi:10.1371/journal.pone.0238642)
Supplement: S3 Table — (DOCX) [file pone.0238642.s003.docx]

|  | **Estimate** | **Standard Error** | **t-value** | **p-value** | **95% Confidence Interval (CI)** |
| --- | --- | --- | --- | --- | --- |
| Reference mean | 5.4636 | 0.4975 | 10.982 | <0.0001 | (4.4778, 6.4494) |
| Intervention^a^ | | | | | |
| Regular spoon | -1.2073 | 0.4318 | -2.796 | 0.0061 | (-2.0629, -0.3517) |
| Special spoon + information | -2.6473 | 0.4318 | -6.131 | <0.0001 | (-3.5029, -1.7917) |
| Information + priming picture | -0.5774 | 0.4318 | -1.337 | 0.1839 | (-1.4330, 0.2782) |
| Information + affect picture | -0.6071 | 0.4318 | -1.406 | 0.1625 | (-1.4627, 0.2485) |
| Canteen^a^ | | | | | |
| A | -0.1332 | 0.4318 | -0.308 | 0.7583 | (-0.9888, 0.7224) |
| C | 0.0230 | 0.4318 | 0.053 | 0.9576 | (-0.8326, 0.8786) |
| D | -0.4527 | 0.4318 | -1.048 | 0.2967 | (-1.3083, 0.4029) |
| E | 2.0085 | 0.4318 | 4.652 | <0.0001 | (1.1529, 2.8641) |
| Weekday^a^ | | | | | |
| Tuesday | 0.5065 | 0.4271 | 1.186 | 0.2382 | (-0.3398, 1.3527) |
| Wednesday | -0.2501 | 0.4271 | -0.586 | 0.5593 | (-1.0963, 0.5961) |
| Thursday | -0.1196 | 0.4271 | -0.280 | 0.7800 | (-0.9658, 0.7266) |
| Friday | 0.6987 | 0.4271 | 1.618 | 0.1085 | (-0.1570, 1.5543) |

^a^Reference categories for each variable used to calculate intra-block effects include: No Intervention/Control; Canteen B; and Monday
