# Supplementary material for: Nudge interventions to reduce fish sauce consumption in Thailand
Source: PLoS One. 2020 Sep 8;15(9):e0238642. doi: 10.1371/journal.pone.0238642 (PMC7478907; doi:10.1371/journal.pone.0238642)
Supplement: S4 Table — (DOCX) [file pone.0238642.s004.docx]

|  | **Estimate** | **Standard Error** | **t-value** | **p-value** | **95% Confidence Interval (CI)** |
| --- | --- | --- | --- | --- | --- |
| Reference mean | 0.2784 | 0.0200 | 13.900 | <0.0001 | (0.2387, 0.3181) |
| Intervention^a^ | | | | | |
| Regular spoon | 0.0161 | 0.0174 | 0.9260 | 0.3563 | (-0.0183, 0.0505) |
| Special spoon + information | 0.0139 | 0.0174 | 0.7970 | 0.4270 | (-0.0206, 0.0483) |
| Information + priming picture | 0.0134 | 0.0174 | 0.7710 | 0.4424 | (-0.0210, 0.0478) |
| Information + affect picture | -0.0018 | 0.0174 | -0.1020 | 0.9188 | (-0.0362, 0.0327) |
| Canteen^a^ | | | | | |
| A | -0.0888 | 0.0174 | -5.1100 | <0.0001 | (-0.1233, -0.0544) |
| C | -0.1044 | 0.0174 | -6.0060 | <0.0001 | (-0.1388, -0.0700) |
| D | -0.0478 | 0.0174 | -2.7500 | 0.0070 | (-0.0822, -0.0133) |
| E | -0.0537 | 0.0174 | -3.0890 | 0.0025 | (-0.0881, -0.0193) |
| Weekday^a^ | | | | | |
| Tuesday | 0.0016 | 0.0172 | 0.0910 | 0.9273 | (-0.0325, 0.0356) |
| Wednesday | 0.0340 | 0.0172 | 1.9760 | 0.0506 | (-0.0001, 0.0680) |
| Thursday | 0.0311 | 0.0172 | 1.8070 | 0.0734 | (-0.0030, 0.0651) |
| Friday | -0.0025 | 0.0172 | -0.1410 | 0.8881 | (-0.0369, 0.0320) |

^a^Reference categories for each variable used to calculate intra-block effects include: No Intervention/Control; Canteen B; and Monday
