# Supplementary material for: Nudge interventions to reduce fish sauce consumption in Thailand
Source: PLoS One. 2020 Sep 8;15(9):e0238642. doi: 10.1371/journal.pone.0238642 (PMC7478907; doi:10.1371/journal.pone.0238642)
Supplement: S5 Table — (DOCX) [file pone.0238642.s005.docx]

|  | **Outcome measurements** | | |
| --- | --- | --- | --- |
|  | **Amount of fish sauce used (grams) per bowl sold per day** | **Amount of fish sauce used (grams) per bowl to which fish sauce was added per day** | **% Bowls to which fish sauce was added per bowl sold per day** |
| R-squared | 0.43 | 0.46 | 0.33 |
| Adjusted R-squared | 0.37 | 0.40 | 0.26 |
| F(12,111) | 6.973 | 7.811 | 4.583 |
| p-value | <0.0001 | <0.0001 | <0.0001 |
